# Analysis of molecular mechanisms of 5-fluorouracil-induced steatosis and inflammation *in vitro* and in mice

**DOI:** 10.18632/oncotarget.14371

**Published:** 2016-12-30

**Authors:** Judith Sommer, Abdo Mahli, Kim Freese, Tobias S. Schiergens, Fulya Suzan Kuecuekoktay, Andreas Teufel, Wolfgang E. Thasler, Martina Müller, Anja K. Bosserhoff, Claus Hellerbrand

**Affiliations:** ^1^ Institute of Biochemistry (Emil-Fischer-Zentrum), Friedrich-Alexander University Erlangen-Nuremberg, Erlangen, Germany; ^2^ Department of Internal Medicine I, University Hospital Regensburg, Germany; ^3^ Biobank o.b. HTCR, Department of General Visceral- and Transplantation Surgery, Ludwig-Maximilians-University Munich, Munich, Germany; ^4^ Comprehensive Cancer Center Erlangen, CCC Erlangen-EMN; Friedrich-Alexander University Erlangen-Nuremberg, Erlangen, Germany

**Keywords:** 5-FU, steatosis, steatohepatitis, mitochondrial dysfunction

## Abstract

Chemotherapy-associated steatohepatitis is attracting increasing attention because it heralds an increased risk of morbidity and mortality in patients undergoing surgery because of liver metastases. The aim of this study was to develop *in vitro* and *in vivo* models to analyze the pathogenesis of 5-fluorouracil (5-FU)-induced steatohepatitis.

Therefore, primary human hepatocytes and HepG2 hepatoma cells were incubated with 5-FU at non-toxic concentrations up to 24 h. Furthermore, hepatic tissue of C57BL/6N mice was analyzed 24 h after application of a single 5-FU dose (200 mg/kg body weight). *In vitro*, incubation with 5-FU induced a significant increase of hepatocellular triglyceride levels. This was paralleled by an impairment of mitochondrial function and a dose- and time-dependently increased expression of fatty acid acyl-CoA oxidase 1 (ACOX1), which catalyzes the initial step for peroxisomal *β*-oxidation. The latter is known to generate reactive oxygen species, and consequently, expression of the antioxidant enzyme heme oxygenase 1 (HMOX1) was significantly upregulated in 5-FU-treated cells, indicative for oxidative stress. Furthermore, 5-FU significantly induced c-Jun N-terminal kinase (JNK) activation and the expression of pro-inflammatory genes IL-8 and ICAM-1. Also *in vivo*, 5-FU significantly induced hepatic ACOX1 and HMOX1 expression as well as JNK-activation, pro-inflammatory gene expression and immune cell infiltration. In summary, we identified molecular mechanisms by which 5-FU induces hepatocellular lipid accumulation and inflammation. Our newly developed models can be used to gain further insight into the pathogenesis of 5-FU-induced steatohepatitis and to develop therapeutic strategies to inhibit its development and progression.

## INTRODUCTION

5-fluorouracil (5-FU) is a uracil analogue and widely used antimetabolite agent for the treatment of various cancer types, including colorectal, breast, and head and neck cancer [1, 2]. 5-FU does not exhibit cytotoxic properties prior to intracellular conversion to one of its active metabolites, namely 5-fluorodeoxyuridine monophosphate (5-FdUMP), 5-fluorodeoxyuridine triphosphate (5-FdUTP) or 5-fluorouridine triphosphate (5-FUTP) [1]. 5-FdUMP is a suicide inhibitor of thymidylate synthase (TYMS), the only cellular *de novo*- source of deoxythymidine monophosphate (dTMP) [1]. Depletion of dTMP leads to deoxynucleotide pool imbalances, resulting in disruption of DNA synthesis and repair [3, 4]. 5-FUTP is misincorporated into RNA, whereby RNA processing and function are impaired [1, 4, 5]. Furthermore, 5-FdUTP incorporation into DNA inhibits DNA synthesis and induces single- and double strand breaks, leading to DNA fragmentation [6–8]. Despite its benefits as a chemotherapeutic agent in neoadjuvant and adjuvant treatment, especially for patients suffering from (metastatic) colorectal cancer, several studies indicate that 5-FU can also induce liver injury [9–17].

Drug-induced liver injury shares several features with non-alcoholic fatty liver disease (NAFLD). In the majority of patients, NAFLD is associated with risk factors reflecting the metabolic syndrome such as obesity, insulin resistance or dyslipidemia. With the continued rise of obesity in the Western countries, the prevalence of NAFLD has followed a similar trend and is today recognized as the most common liver disease worldwide [18, 19]. NAFLD is characterized by hepatic steatosis, which is also the most documented liver pathology observed in association with 5-FU administration [14, 16, 17, 20]. In general, hepatic steatosis is characterized by intravesicular accumulation of fat in the form of triglycerides within hepatocytes [18, 19]. This is due to an imbalance between lipid uptake on the one hand and their combustion or secretion on the other hand [18]. In NAFLD, “simple” steatosis can progress to non-alcoholic steatohepatitis (NASH), a more serious liver condition characterized by death of hepatocytes and liver inflammation with and without hepatic fibrosis [18, 19].

Generally, the presence of steatosis renders the liver more susceptible to hepatic injury [21]. Oxidative stress, mitochondrial dysfunction and cytokine induction have been identified as further factors that sensitize the liver to an inflammatory reaction [18, 19].

Several studies showed that 5-FU induces oxidative stress in cancer cells [22–24]. Therefore, 5-FU might also be able to promote the development of steatohepatitis in the presence of steatosis. Indeed, the so-called chemotherapy-associated steatohepatitis (CASH) is frequently observed in patients treated with combination therapies of 5-FU and irinotecan [9–13]. Although this damage is mostly ascribed to irinotecan, an (additional) pathological role of 5-FU cannot be excluded. To date, the capability of 5-FU to induce CASH has not been investigated in detail. Thus, the aim of this study was to develop *in vitro* and *in vivo* models for 5-FU-induced steatohepatitis and to identify the underlying mechanisms for the induction of steatosis and its progression to inflammation.

## RESULTS

### Effect of 5-FU on hepatocellular viability and lipid accumulation *in vitro*

**Figure 1:**
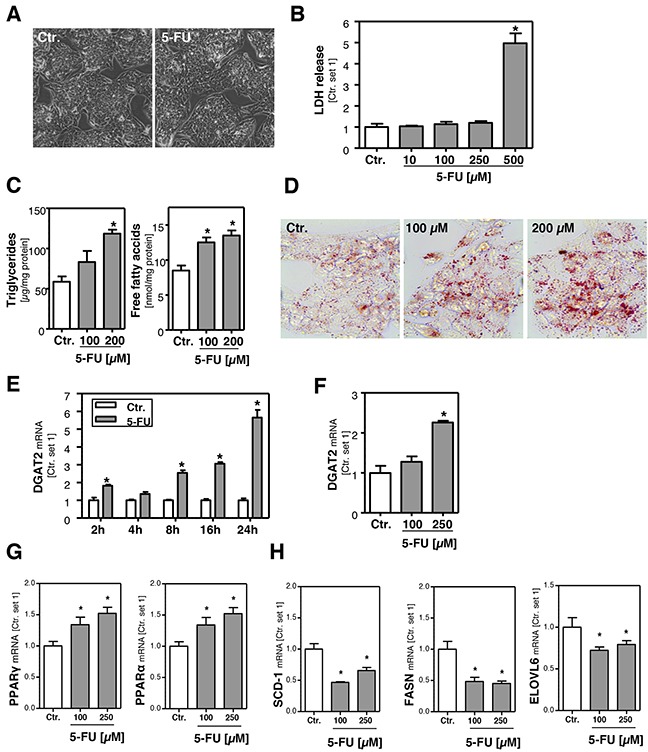
Effect of 5-FU on hepatocellular viability and lipid accumulation **A**. Microscopic images of HepG2 cells incubated with 5-FU (250 μM) for 24 h and control cells. **B**. Quantification of LDH release into the supernatant of HepG2 cells treated with different 5-FU doses as indicated. **C**. Analysis of intracellular triglycerides and free fatty acids content normalized to total cellular protein. **D**. Oil red O staining of HepG2 cells incubated with 5-FU for 24 h and control cells. Furthermore, HepG2 cells were incubated with 5-FU (200 μM) for different times as indicated. Moreover, cells were incubated with two different 5-FU doses for 24 h. Analysis of mRNA levels of **E, F**. DGAT2, **G**. PPARγ and PPARα and **H**. SCD-1, FASN and ELOVL6 by quantitative RT-PCR (*: p<0.05 compared to control).

Initially, we determined the dose range in which 5-FU did not affect viability of HepG2 cells. Microscopic analysis (Figure 1A) and assessment of LDH levels in the supernatant (Figure 1B) showed that concentrations up to 250 μM 5-FU were non-toxic for HepG2 cells. In response to this 5-FU dose TYMS expression was significantly increased (Supplementary Figure 1A) while expression levels of dihydropyrimidine dehydrogenase (DPD), the 5-FU metabolizing enzyme, did not significantly change (Supplementary Figure 1B). It has been described that expression of genes encoding enzymes relevant to 5-flurouracil metabolism correlates with histologically diagnosed chemotherapy-induced hepatic injury [25]. The missing increase of DPD in our experimental setting may be explained by the application of non-toxic 5-FU doses. However, in this dose range, incubation with 5-FU significantly increased intracellular free fatty acid (FFA) and triglyceride (TG) levels (Figure 1C). Compared to control cells, FFA and TG content approximately doubled in the presence of 200 μM 5-FU. This finding was supported by oil red O staining, showing microvesicular steatosis in 5-FU-treated cells (Figure 1D).

In line with these results, expression levels of diacylglycerol acyltransferase 2 (DGAT2), which catalyzes the terminal step in the formation of triglycerides, were time- and dose-dependently increased in response to 5-FU (Figure 1E, 1F). Interestingly, 5-FU also caused a significant induction of peroxisome proliferator activated receptor alpha (PPAR-α) and PPAR-γ, both critical regulators of hepatic lipid metabolism (Figure 1G). However, expression levels of stearoyl-Coenzyme A desaturase 1 (SCD-1) and fatty acid synthase (FASN) were not increased but even lower in 5-FU-treated compared to untreated cells (Figure 1H). Also expression of fatty acid elongases 6 (ELOVL6), which has been described as critical promoter of nonalcoholic steatohepatitis [26], was reduced by 5-FU (Figure 1H). Together, these findings indicate that the 5-FU-induced FFA and TG accumulation in HepG2 cells is not mediated via *de novo* lipogenesis.

### Effect of 5-FU on *β*-oxidation and oxidative stress in hepatoma cells *in vitro*

**Figure 2:**
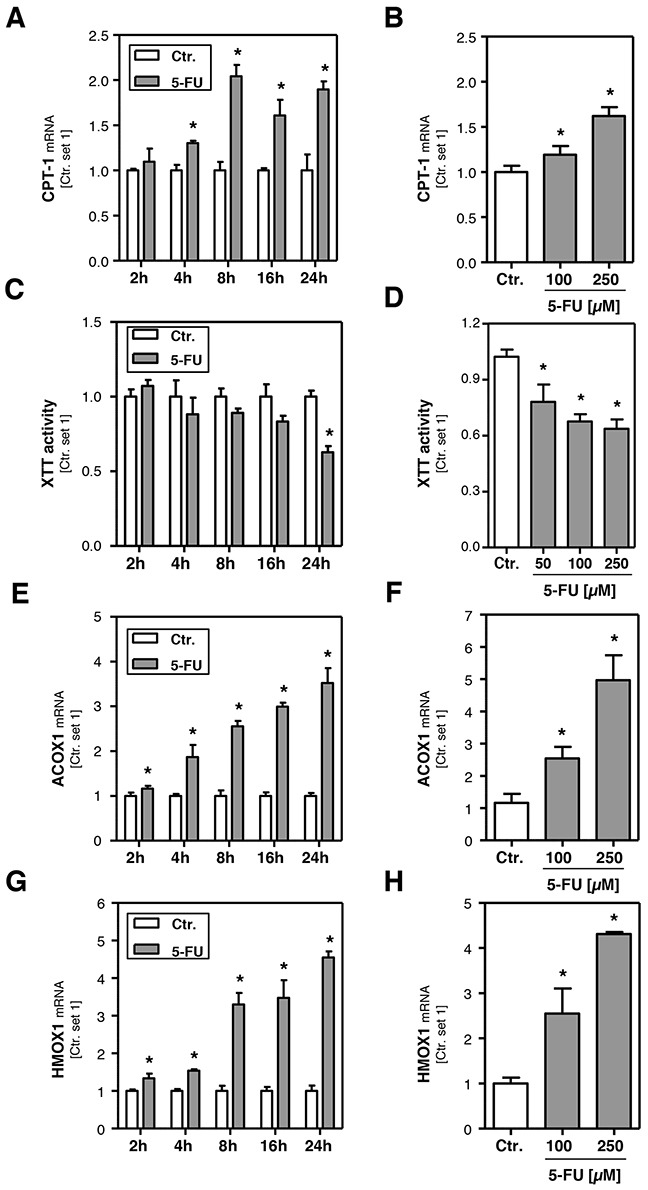
Effect of 5-FU on hepatocellular *β*-oxidation and oxidative stress HepG2 cells were incubated with 5-FU (200 μM) for different times as indicated. Furthermore, cells were incubated with two different 5-FU doses for 24 h. Analysis of mRNA levels of **A, B**. CPT-1, **E, F**. ACOX1 and **G, H**. HMOX1 by quantitative RT-PCR. **C, D**. Analysis of XTT activity. (*: p<0.05 compared to control).

In search for the underlying mechanisms of 5-FU induced lipid accumulation, we analyzed expression levels of genes that are involved in combustion of fatty acids. Carnitine palmitoyltransferase-1 (CPT-1) is the key enzyme for the transport of fatty acids into mitochondria for *β*-oxidation [27, 28]. 5-FU caused a dose-dependent induction of CPT-1 expression in HepG2 cells (Figure 2B), whereby the maximum was reached 8 h after stimulation (Figure 2A). ATP-citrate lyase (ACLY) has been described as a regulator of the carnitine system [29], however, also this lipogenic enzyme was downregulated in response to 5-FU treatment (Supplementary Figure 2A, 2B). Interestingly, the increased CPT-1-expression was paralleled by reduced mitochondrial function as analyzed by XTT-assays (Figure 2C, 2D). Impairment of mitochondrial fatty acid combustion can lead to the activation of extra-mitochondrial pathways. Indeed, 5-FU-induced the expression of fatty acid acyl-CoA oxidase 1 (ACOX1) (Figure 2E, 2F). This enzyme catalyzes the initial step for peroxisomal *β*-oxidation [28]. The 5-FU-induced ACOX1 expression continued to increase beyond the observed time period of 24 h (Figure 2E) and resulted in approximately 4-fold higher expression levels after 24 h stimulation (Figure 2F). Peroxisomal *β*-oxidation is known to lead to the production of reactive oxygen species (ROS) [27, 30–32], and the antioxidant enzyme heme oxygenase 1 (HMOX1) has been shown to be increased in response to oxidative stress [33]. Stimulation with 5-FU caused a time- and dose-dependent induction of HMOX1 expression levels (Figure 2G, 2H) that paralleled the pattern of 5-FU effects on ACOX1 mRNA levels. Together these data suggest that in response to 5-FU-induced lipid accumulation, mitochondrial and peroxisomal *β*-oxidation are up-regulated and thus induce oxidative stress, a known inducer of an inflammatory response.

### Effect of 5-FU on hepatocellular inflammatory response *in vitro*

**Figure 3:**
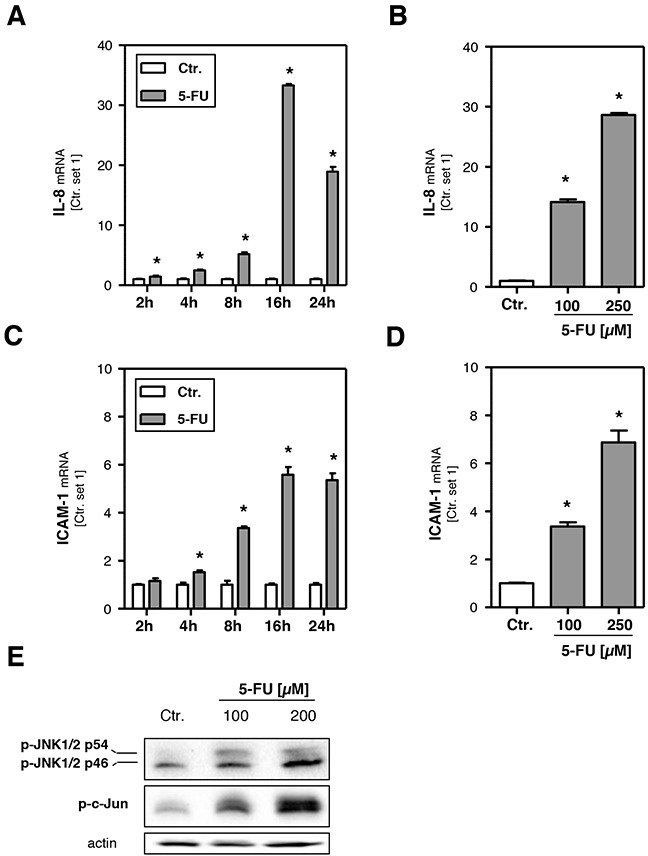
Effects of 5-FU on hepatocellular inflammatory response HepG2 cells were incubated with 5-FU (200 μM) for different times as indicated and for 24 h with two different doses, respectively. Analysis of mRNA levels of **A, B**. IL-8 and **C, D**. ICAM-1 by quantitative RT-PCR (*: p<0.05 compared to control). **E**. Western Blot analysis of p-JNK1/2 and p-c-Jun protein levels of HepG2 cells incubated with 5-FU for 12 h. Actin protein levels were measured to ensure equal loading.

To investigate the potential of 5-FU to induce an inflammatory response the expression levels of interleukin-8 (IL-8), a cytokine involved in the initiation of an inflammatory reaction [34], and intercellular adhesion molecule-1 (ICAM-1), a cell surface molecule that facilitates the adhesion of leukocytes [34], were analyzed. Both IL-8 (Figure 3A, 3B) and ICAM-1 (Figure 3C, 3D) expression levels were markedly increased in response to 5-FU. Expression levels showed a time-dependent increase as early as 2 h after 5-FU treatment and reached a plateau at 16 h (Figure 3A, 3C). Expression of IL-8 and ICAM-1 is dependent on c-Jun N-terminal kinase (JNK) activity [35, 36], and increased JNK activation has also been identified as critical pathological factor in the progression from steatosis to inflammation in NAFLD [37]. Western blot analysis revealed that also 5-FU treatment significantly induced levels of phospho-c-Jun N-terminal kinase 1/2 (phospho-JNK1/2) and phosphorylated c-Jun (Figure 3E). These data suggest that activation of the JNK pathway is mediating 5-FU-induced pro-inflammatory gene expression in hepatoma cells *in vitro*.

### Effect of 5-FU on primary human hepatocytes *in vitro*

**Figure 4:**
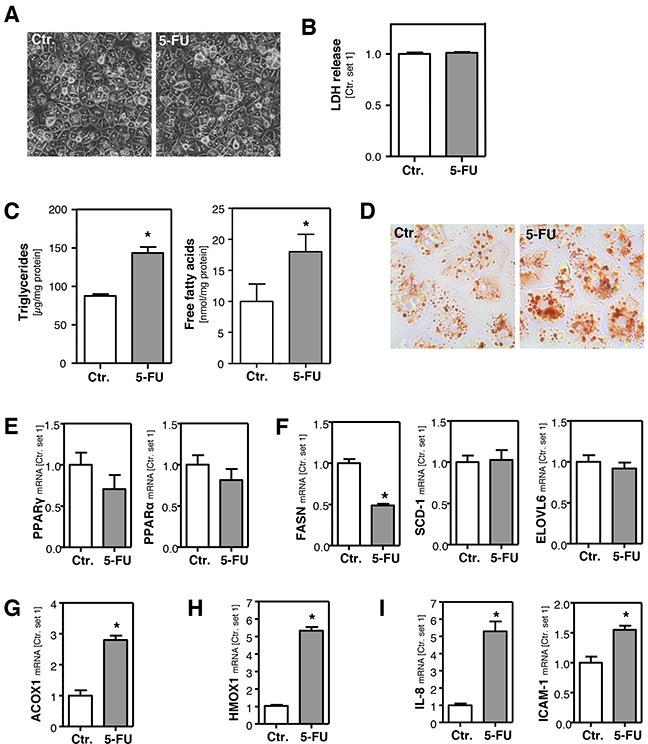
Effect of 5-FU in primary human hepatocytes Primary human hepatocytes were incubated with 5-FU (500 μM) for 24 h. **A**. Microscopic images. **B**. Quantification of LDH release into the supernatant. **C**. Analysis of intracellular triglycerides and free fatty acids content normalized to total cellular protein. **D**. Oil red O staining. Analysis of mRNA levels of **E**. PPARγ and PPARα, **F**. FASN, SCD-1 and ELOVL6, **G**. ACOX1, **H**. HMOX1 and **I**. IL-8 and ICAM-1 by quantitative RT-PCR (*: p<0.05 compared to control).

HepG2 cells are a widely used cellular *in vitro* model to analyze drug metabolism and toxicity. Still, we wanted to verify observed 5-FU effects in primary human hepatocytes (PHH). A 5-FU dose of 500 μM did not affect the viability of PHH (Figure 4A, 4B), and we used this concentration for subsequent *in vitro* analyses in PHH. 5-FU stimulation did not affect DPD expression levels in PHH (Supplementary Figure 3A), which were similar as in HepG2 cells (Supplementary Figure 3B). Moreover, also TYMS expression was not altered in primary PHH in response to 5-FU treatment (Supplementary Figure 3C). This difference to HepG2 cells may be explained by the fact that expression levels of this 5-FU target enzyme were almost absent in PHH as compared to HepG2 hepatoma cells (Supplementary Figure 3D). However, also in PHH, 5-FU caused a significant increase of the intracellular FFA and triglyceride level (Figure 4C, 4D). PPAR-α and PPAR-γ expression levels were not significantly altered by 5-FU treatment in PHH (Figure 4E) and also SCD-1, FASN, ACLY and ELOVL6 expression were similar or lower in 5-FU stimulated PHH compared to control cells (Figure 4F and Supplementary Figure 2C). These findings indicated that also in PHH 5-FU-induced steatosis is not mediated via *de novo* lipogenesis. However, incubation with 5-FU significantly induced ACOX1 and HMOX1 expression (Figure 4G, 4H), as well as expression of pro-inflammatory genes (Figure 4I) in PHH. In summary, the 5-FU effects on PHH reveal a high congruence with the data obtained with HepG2 cells and indicate that this human cell line is a suitable *in vitro* model to study 5-FU-mediated effects related to hepatocellular steatosis and inflammation.

### Effect of 5-FU on hepatic steatosis and inflammation in mice

**Figure 5:**
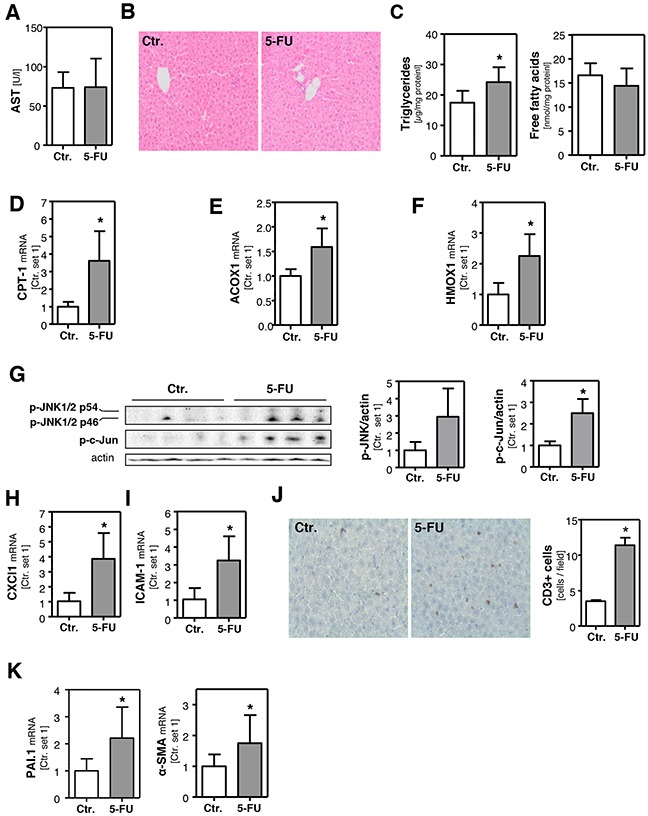
Effect of 5-FU on hepatic steatosis and inflammation in mice Mice were intraperitoneally injected with a single dose 5-FU (200 mg/kg) and liver tissue samples were collected 24 h after injection. Control mice were injected with solvent isotonic saline solution. **A**. Plasma levels of aspartate transaminase (AST). **B**. H/E staining of liver tissue samples. **C**. Hepatic triglycerides and free fatty acids content normalized to total protein. Analysis of mRNA levels of **D**. CPT-1, **E**. ACOX1 and **F**. HMOX1 by quantitative RT-PCR. **G**. Western blot analysis of hepatic p-JNK and p-c-Jun protein levels (left panel). Densitometric analysis of p-JNK/actin ratio (middle panel) and p-c-Jun/actin ratio (right panel). Analysis of mRNA levels of **H**. CXCl1 and **I**. ICAM-1 by quantitative RT-PCR. **J**. CD3 staining of liver tissue samples (left panel). Quantification of CD3-positive cells (right panel). Analysis of mRNA levels of **K**. PAI-1 and α-SMA by quantitative RT-PCR (*: p<0.05 compared to control).

Next, we wanted to verify our *in vitro* findings in an *in vivo* model of 5-FU-induced steatohepatitis in mice. For that, mice were intraperitoneally injected with a single dose of 5-FU (200 mg/kg body weight) and liver tissue and blood samples were collected 24 h after injection. Plasma aspartate transaminase (AST) levels were not elevated (Figure 5A) and also H/E staining of liver tissue did not show significant histological pathologies (Figure 5B) in 5-FU-treated mice under this experimental conditions. As observed in PHH, DPD and TYMS expression were not altered in the livers of 5-FU-treated mice (Supplementary Figure 4A). Also FFA levels did not significantly differ, but triglyceride levels were significantly elevated in the livers of 5-FU-treated mice (Figure 5C). In contrast,PPAR-α and PPAR-γ expression levels were not significantly altered by 5-FU treatment (Supplementary Figure 4B), and SCD-1, FASN, ACLY and ELOVL6 expression was similar or lower in the livers of 5-FU-treated mice compared to control animals (Supplementary Figure 4C). These findings indicated that also *in vivo*, 5-FU-induced steatosis is not mediated via *de novo* lipogenesis. However, both hepatic CPT-1 (Figure 5D) and ACOX1 expression (Figure 5E) were significantly induced by 5-FU. Furthermore, hepatic HMOX1 expression was significantly higher in 5-FU-treated mice compared to controls (Figure 5F), indicative for increased oxidative stress. Moreover, 5-FU caused an induction of the JNK pathway in the liver of treated mice (Figure 5G). Furthermore, hepatic CXCl1 and ICAM-1 expression were significantly induced (Figure 5H, 5I) and also immunohistological CD3 staining indicated an inflammatory response in the liver of 5-FU-treated mice (Figure 5J). Pro-ininflammatory cytokines and hepatic inflammation, respectively, have been suggested as regulators of hepatic plasminogen activator inhibitor-1 (PAI-1) expression. Accordingly, we observed a significantly increased expression of this serine protease inhibitor in the livers of 5-FU-treated mice (Figure 5K). Results of studies performed in animal models of alcoholic and non-alcoholic fatty liver disease suggest that PAI-1 is a key modulator of hepatic lipid transport and also contributes to hepatic inflammation and fibrosis [38]. Actually, alpha-smooth muscle actin (α-sma) expression (Figure 5K) was significantly increased in the livers of 5-FU-treated mice indicating the activation of hepatic stellate cells (HSC), a key event of hepatic fibrosis [39]. Although the expression levels of transforming growth factor-β (TGF-β) and collagen I were not yet significantly elevated (data not shown), repeated and long-term application of 5-FU may also induce hepatic fibrosis in mice similarly as observed in patients receiving long-term chemotherapy [40].

In summary, the *in vivo* findings were consistent with the observations made *in vitro*. Expression of enzymes involved in mitochondrial and peroxisomal *β*-oxidation was significantly induced and paralleled by an upregulation of the antioxidant enzyme HMOX1, indicative for oxidative stress in response to 5-FU treatment. Moreover, 5-FU induced JNK-activation, hepatocellular steatosis and an inflammatory reaction in both models.

## DISCUSSION

The aim of this study was to investigate molecular mechanisms by which 5-FU affects hepatic steatosis and inflammation.

Once, we established an *in vitro* model applying HepG2 cells. This cell line has been most extensively employed in many studies analyzing drug metabolism and toxicity, since the cells retain a large part of cellular functions similar to those of normal hepatocytes [41, 42]. Also because of the high degree of morphological and functional differentiation, HepG2 cells are a suitable model to study intracellular trafficking and drug targeting *in vitro* [43]. In the present study, we found that 5-FU-mediated effects in HepG2 cells and in primary human hepatocytes were very similar. Therefore, we propose that also for future studies analyzing 5-FU-mediated effects on hepatocytes, such as screening of drugs that interfere with the observed pathological mechanisms, respectively, HepG2 can be a suitable model. 5-FU was applied in a dose range not causing cellular injury to focus on direct, cell specific effects. The applied 5-FU doses were in line with previous studies of Novak *et al*. [44] and Gajski *et al*. [45], who also found that doses up to 250 μM are non-toxic for HepG2 cells. Also for analyzing the effect of 5-FU in primary human hepatocytes, a dose was chosen in accordance with previous studies showing that it does not affect hepatocellular viability [46]

For *in vivo* analyses, we administered a single dose of 200 mg/kg 5-FU to mice. This dose was below LD50 determined earlier in mice (250-500 mg/kg, depending on the time of day) [47] and in line with previous studies analyzing the anti-tumorigenic effect of 5-FU in murine cancer models [48]. Also here, we chose experimental conditions not causing significant hepatocellular injury to specifically address direct 5-FU effects on liver cells. And we applied only a single 5-FU dose to focus on early 5-FU-mediated effects.

An important observation in our study was that 5-FU treatment induced a significant triglyceride accumulation in hepatocytes and HepG2 cells, an incidence known from clinical trials, where up to half of patients receiving 5-FU monotherapy were found to develop hepatic steatosis [14–17]. One factor contributing to hepatocellular lipid accumulation is endogenous *de novo* lipogenesis [49, 50]. However, key enzymes of hepatic lipogenesis were not significantly increased or even downregulated, respectively, in response to 5-FU treatment. Another possible trigger for lipid accumulation is impaired fatty acid degradation. Generally, fatty acid molecules are broken down *via* beta-oxidation in the mitochondria to generate acetyl-CoA. However, steatohepatitis-inducing drugs are often inhibitors of the mitochondrial respiratory chain [51] and also 5-FU has been shown to be related to a reduction of mitochondrial membrane potential and mitochondrial membrane collapse [52]. Fitting to this, we observed a dose- and time- dependent impairment of mitochondrial function in response to 5-FU treatment. An impairment of the respiratory chain leads to an accumulation of electrons, which directly react with oxygen to produce harmful ROS [51]. Furthermore, lack of cofactors for *β*-oxidation such FAD and NAD^+^ may additionally decrease metabolism of FFAs, which accumulate and thus induce CPT-1 mRNA expression [53]. Fitting to this, induction of CPT-1 expression was also observed in the livers of mice after acute inhibition of mitochondrial *β*-oxidation [54]. Thus, it appears that also increased CPT-1 expression in 5-FU-treated cells and livers was caused by impaired mitochondrial *β*-oxidation.

A consequence of impaired mitochondrial *β*-oxidation is a shift to extra-mitochondrial pathways. In line with this, we observed a significant upregulation of ACOX1, the initial enzyme for peroxisomal *β*-oxidation. Also in rodent models of (non-alcoholic) steatosis peroxisomal *β*-oxidation was shown to be increased [55]. This form of FFA combustion causes increased hydrogen peroxide generation, thus contributing to harmful ROS production in addition to an impaired respiratory chain. Also in our *in vitro* and *in vivo* models, 5-FU caused a marked upregulation of HMOX1 expression indicative for oxidative stress. This is in accordance with other studies that showed that 5-FU induces oxidative stress in cancer cells [22, 56]. Our *in vitro* data indicate that the 5-FU-induced shift to peroxisomal *β*-oxidation and consecutive induction of oxidative stress and inflammation occur already after several hours. Accordingly, we also found increasing ACOX1 expression, elevated HMOX1 and pro-inflammatory gene expression as well as CD3-infiltration (Supplementary Figure 5A-5F) *in vivo* in mouse livers already 12 h after 5-FU application.

Taken together, we propose the hypothesis that the observed triglyceride accumulation induced by 5-FU is most likely the result of mitochondrial *β*-oxidation impairment, possibly by affecting the respiratory chain. Although fatty acids are progressively oxidized in peroxisomes, they are insufficiently metabolized and accumulate. Together, impaired mitochondrial and enhanced peroxisomal fatty acid metabolism lead to oxidative stress.

ROS are known inducers of pro-inflammatory genes [30, 31], and indeed we observed increased IL-8 and ICAM-1 expression as well as induced hepatic infiltration with immune cells in response to 5-FU treatment. Moreover, 5-FU treatment led to a significant activation of JNK. This stress-activated protein kinase is an important modulator in response to liver injury [57], and we and others have shown that it plays a critical role in the development and progression of hepatic inflammation in alcoholic as well as non-alcoholic fatty liver disease (NAFLD) [37, 58]. Obesity is the major risk factor for NAFLD and it is also an important risk factor for different types of cancer [59]. Therefore, it is likely that a significant number of cancer patients receiving chemotherapy have also NAFLD. Future experimental and epidemiological studies are warranted to study whether already present hepatic steatosis is a trigger for more hepatic inflammation and injury. Furthermore, hepatic JNK-activation and inflammation may play a crucial role in the progression and development of hepatocellular cancer (HCC) [60, 61] as well as chemotherapy resistance of HCC cells [62, 63]. Moreover, hepatic inflammation and expression of IL-8 have been shown to promote hepatic metastasis of several types of cancers [64, 65]. Therefore, it is intriguing to speculate whether our findings may also have impact on progression and chemotherapy (resistance) of primary and secondary liver cancer, which needs to be addressed in future studies.

In summary, our study indicates a crucial role of 5-FU in the development of chemotherapy-associated steatohepatitis. As pointed out in the introduction, a two hit model for the development and progression of non-alcoholic fatty liver disease has been proposed [18, 66]. Critical steps are hepatic steatosis and its progression to steatohepatitis. In this study, we identify molecular mechanisms by which 5-FU can induce both hepatocellular lipid accumulation and inflammatory gene expression. Herewith, 5-FU alone as well as together with other factors, such as irinotecan, may be responsible for the critical hit tipping the balance towards hepatic steatosis or from steatosis to steatohepatitis, respectively. Our newly developed *in vitro* and *in vivo* models can be used to get further insight into the pathogenesis of 5-FU-induced steatohepatitis and to develop therapeutic strategies to inhibit the pathological effects of this chemotherapeutic drug on hepatocytes.

## MATERIALS AND METHODS

### Cells and cell culture model

Isolation and culture of primary human hepatocytes (PHH) were performed as described [67, 68]. Human liver tissue for cell isolation was obtained from the charitable state controlled foundation HTCR [69], with informed patient consent and approved by the local Ethics Committee. HepG2 hepatoma cells (ATCC No. HB-8065) were cultured as described [70].

For stimulation experiments with 5-FU, cells were seeded in standard 6-well-plates at a density of 5×10^5^ cells/well and were allowed to adhere for 24 h before treatment. Cytotoxic effects were monitored by analysis of LDH release into the supernatant (cytotoxicity detection kit; Roche Applied Sciences, Indianapolis, IN).

### Murine models of 5-FU induced steatohepatitis

Female C57BL/6N mice (n=5/group), aged 7-9 weeks, were intraperitoneally injected with a single dose 5-FU (200 mg/kg) or isotonic saline solution (control group). Liver tissue and blood samples were collected 12 h or 24 h after injection and immediately frozen and stored at -80°C until subsequent analyses.

### Analysis of cellular lipid content

Cellular lipid droplets were visualized by Oil Red O staining as described [71]. Furthermore, total free fatty acids (FFA) and cellular triglycerides were extracted and quantified with the free fatty acids kit (half micro test) (Roche diagnostics, Mannheim, Germany) and the triglyceride determination kit (GPO) (Sigma, Deisenhofen, Germany) [71].

### Mitochondrial activity assay

For quantification of hepatocellular mitochondrial activity, the colorimetric XTT assay (Roche Diagnostics, Mannheim, Germany) was used according to the manufacturer's instructions.

### Quantitative real-time-PCR analysis

RNA isolation from cells and tissues and subsequent reverse transcription were performed as described [72]. Quantitative real-time-PCR was performed applying LightCycler technology (Roche) [72]. The following sets of primers were used: human ACLY (forward: 5′-CGGACTTCGGCAGAGGTAGA-3′, reverse 5′-GGG GAG GGA ACT CGA TGT CA-3′), human DPD (forward: 5′-AGG ACG CAA GG AGG GTT TG-3′, reverse: 5′-GTC CGC CGA GTC CTT ACT GA-3′), human ELOVL6 (forward: 5′-CCA GTC AAC TCC TCG CAC TTT-3′, reverse: 5′-TGA CCG TGT CCG GTA TTT CC-3′), human HMOX1 (forward: 5′-GAG TGT AAG GAC CCA TCG GA-3′, reverse 5′-GCC AGC AAC AAA GTG CAA G-3′), human ICAM-1 (forward: 5′-CTG TCA CTC GAG ATC TTG AGG-3′, reverse: 5′-CCT GCA GTG CCC ATT ATG A-3′), human IL-8 (forward: 5′-TCT GCA GCT CTG TGT GAA GGT GCA GTT-3′, reverse: 5′-AAC CCT CTG CAC CCA GTT TTC CT-3′), human TYMS (forward: 5′-CCT CTG CTG ACA ACC AAA CG, reverse: 5′-GAA GAC AGC TCT TTA GCA TTT G-3′); murine ACLY (forward: 5′-GCA CCC AGA AGG CAA GAT CC-3′, reverse: 5′-CTT GGG ACT GAA TCT TGG GGC-3′), murine DPD (forward: 5′-TCT CAG CCT ACA ATG CCC CT-3′, reverse: 5′-GTT GTC CCC CGG ATG ATT CTT-3′), murine ELOVL6 (forward: 5′-AGA ACA CGT AGC GAC TCC GA-3′, reverse: 5′-AGC GTA CAG CGC AGA AAA CA-3′) and murine TYMS (forward: 5′-GAC TGC TCC GTT ATG CTG GTG-3′, reverse: 5′-CGT TTG GTT GTG AGC AGA GGA-3′). Other human and murine mRNA expression analyses were performed using QuantiTect Primer Assays according to the manufacturer's instructions (Qiagen, Hilden, Germany). Amplification of cDNA derived from human and murine 18S rRNA (forward: 5′-AAA CGG CTA CCA CAT CCA AG-3′, reverse: 5′-CCT CCA ATG GAT CCT CGT TA-3′) was used for normalization.

### Protein analysis

Protein extraction and Western blotting were performed as described [73] applying the following primary antibodies: rabbit polyclonal anti-phospho-JNK (#9251) and rabbit monoclonal anti-phospho-c-Jun (#3270) from Cell Signaling Technology (Danvers, MA, USA; all diluted 1:1,000), and mouse monoclonal anti-actin (Merck Millipore, Billerica, MA, USA; MAB1501, 1:10,000). Goat anti-rabbit (Santa Cruz Biotechnology, Heidelberg, Germany; sc-2030, 1:2,000) and goat anti-mouse (Santa Cruz Biotechnology, sc-2005, 1:3,000) were used as secondary antibody.

### Histological and immunohistological analyses

For hematoxylin and eosin (HE) staining and immunohistochemical analysis, standard 5 μm sections of formalin-fixed and paraffin-embedded tissue blocks were used. Immunohistochemical CD3 staining was performed using a rabbit anti-human T cell CD3 peptide antibody (Sigma, Saint Louis, Missouri, USA) as described [37].

### Statistical analysis

Values are presented as mean ± SEM or as mean ± SD in case of *in vivo* experiments. Comparison between groups was made using the Student's unpaired t-test. A p value <0.05 was considered statistically significant. All calculations were performed using the statistical computer package GraphPad Prism version 5.00 for Windows (GraphPad Software, San Diego, USA).

Microscopical images were taken using an Olympus™ CKX41 microscope with the ALTRA 20 Soft Imaging System™ and Cell^A^ software version 2.6 (Olympus Soft Imaging Solutions GmbH, Münster, Germany). Images were processed using IrfanView™ software version 4.36 (Irfan Skiljan, Jajce, Bosnia).

## SUPPLEMENTARY FIGURES


