# Supplementary material for: Analysis of molecular mechanisms of 5-fluorouracil-induced steatosis and inflammation in vitro and in mice
Source: Oncotarget. 2016 Dec 30;8(8):13059–72. doi: 10.18632/oncotarget.14371 (PMC5355077; doi:10.18632/oncotarget.14371)
Supplement: Supplementary file 1 [file oncotarget-08-13059-s001.pdf]

Analysis of molecular mechanisms of 5-fluorouracil-induced steatosis and inflammation *in vitro* and in mice

SUPPLEMENTARY FIGURES

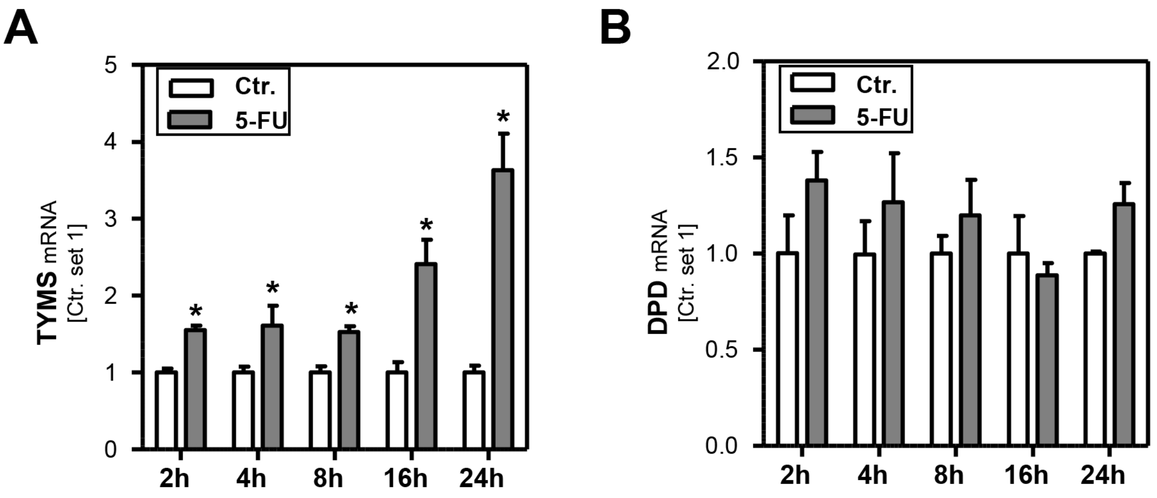

Supplementary Figure 1: Effect of 5-FU on expression of metabolizing enzymes. HepG2 cells were incubated with 5-FU (200 µM) for different times as indicated. Analysis of mRNA levels of **A.** TYMS and **B.** DPD by quantitative RT-PCR (*: $p<0.05$ compared to control).

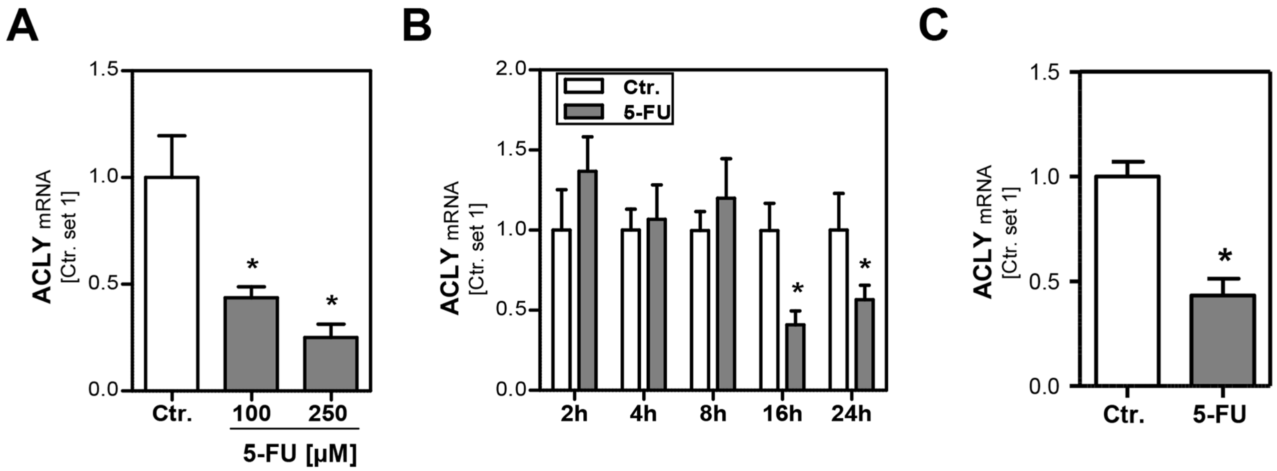

Supplementary Figure 2: Effect of 5-FU on ACLY expression. Analysis of mRNA levels of ACLY by quantitative RT-PCR. **A.** HepG2 cells were incubated with 5-FU for 24h. **B.** HepG2 cells were incubated with 5-FU (200 µM) for different times as indicated. **C.** Primary human hepatocytes were incubated with 5-FU (500 µM) for 24 h(*: $p<0.05$ compared to control).

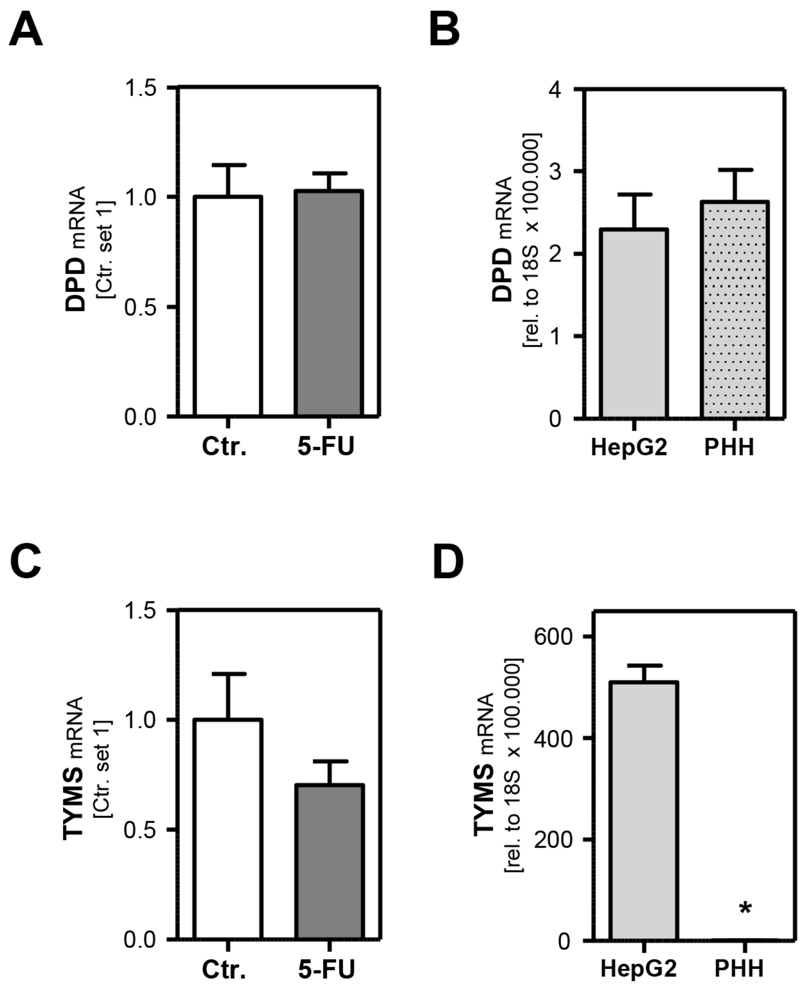

Supplementary Figure 3: Effect of 5-FU on DPD and TYMS expression in primary human hepatocytes and comparison of expression levels with HepG2 cells. PHH were incubated with 5-FU (500 µM) for 24h. Analysis of mRNA levels of **A.** DPD and **C.** TYMS by quantitative RT-PCR. Comparison of expression levels of **B.** DPD and **D.** TYMS relative to 18S mRNA in untreated HepG2 cells versus untreated PHH. (*: $p<0.05$ compared to HepG2).

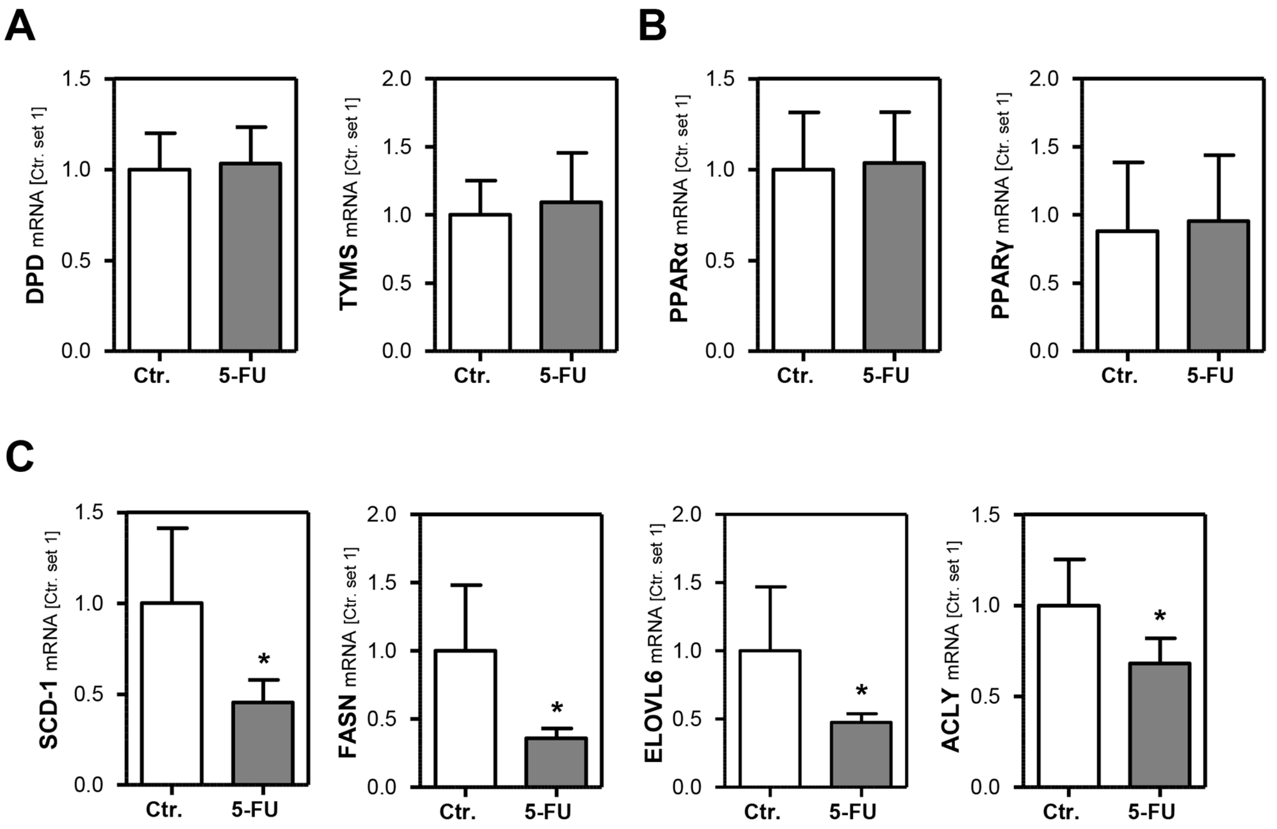

Supplementary Figure 4: Effect of 5-FU on 5-FU-metabolizing enzymes and de novo-lipogenesis in mice. Mice were intraperitoneally injected with a single dose 5-FU (200 mg/kg) and liver tissue samples were collected 24 h after injection. Control mice were injected with solvent isotonic saline solution. Analysis of mRNA levels of **A.** DPD and TYMS, **B.** PPARα andPPARγ and **C.** FASN, SCD-1, ELOVL6 andACLY by quantitative RT-PCR (*: p<0.05 compared to control).

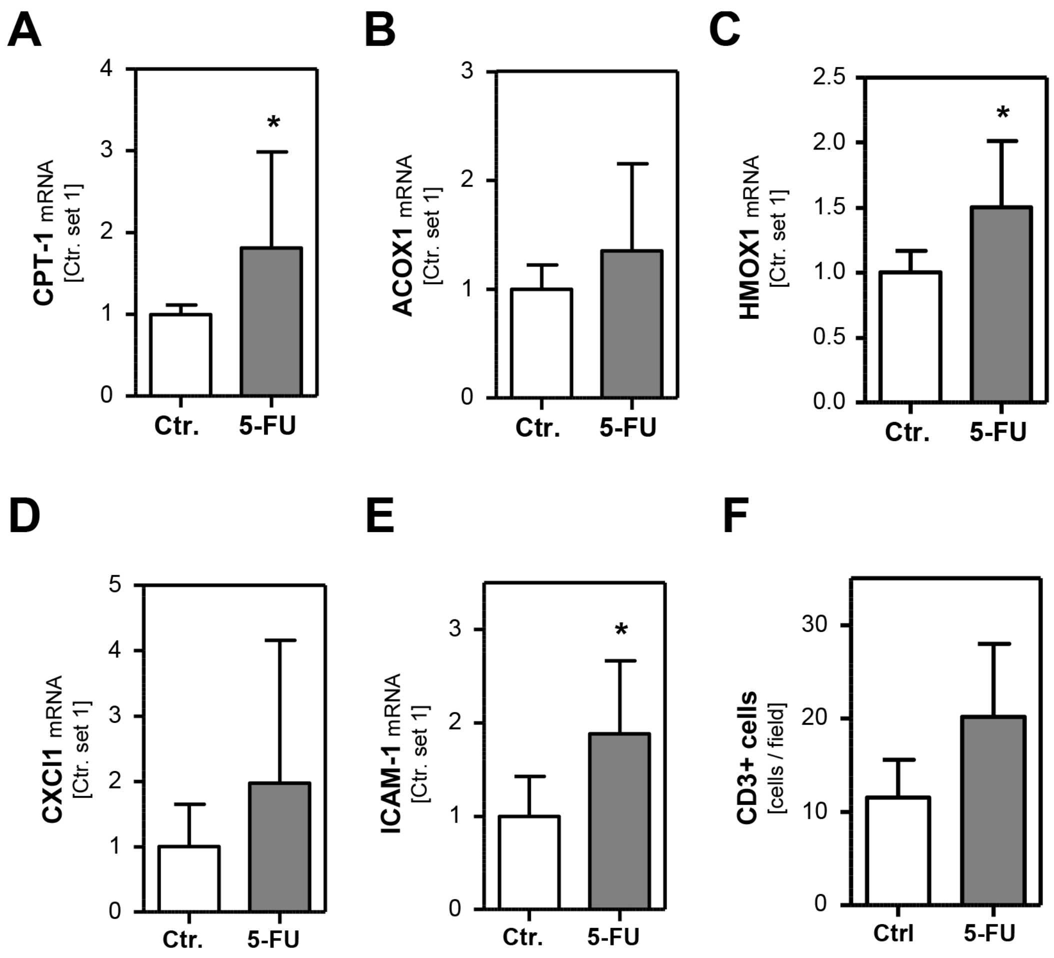

Supplementary Figure 5: Effect of 5-FU on hepatic β-oxidation, oxidative stress and inflammation in mice. Mice were intraperitoneally injected with a single dose 5-FU (200 mg/kg) and liver tissue samples were collected 12 h after injection. Control mice were injected with solvent isotonic saline solution. Analysis of mRNA levels of **A.** CPT-1, **B.** ACOX1, **C.** HMOX1, **D.** CXCl1 and **E.** ICAM-1 by quantitative RT-PCR (*: $p<0.05$ compared to control). **F.** Quantification of CD3-positive cells.
